# Age-specific reference intervals for serum IgG, IgA, and IgM in healthy Brazilian children and adolescents

**DOI:** 10.3389/fimmu.2026.1969584

**Published:** 2026-10-13

**Authors:** Cristina Frias Sartorelli Toledo Piza, Carolina Sanchez Aranda, Ligia Maria de Oliveira Machado, Celso Mendanha, Maria Candida Faria Varanda Rizzo, Rafaela Rola Leite Guimaraes, Mariana Pimentel Gouveia-Pereira, Fernanda Aimée Nobre, Ana Carolina da Matta Ain, Antonio Condino-Neto

**Affiliations:** 1 Department of Pediatrics, Federal University of Sao Paulo (Unifesp), Sao Paulo, Brazil; 2 Department of Pediatrics, University of Taubate, Taubate, Brazil

**Keywords:** immunoglobulins, pediatrics, primary immunodeficiencies, quantile regression, reference intervals

## Abstract

**Background:**

Serum immunoglobulin reference intervals vary by age, ethnicity, and environment, limiting the clinical utility of international guidelines for Brazilian pediatric patients. This study established age-specific reference intervals for serum IgG, IgA, and IgM concentrations in healthy Brazilian children and adolescents, focusing on defining precise trajectories across distinct developmental stages.

**Methods:**

Serum samples from 2,911 healthy individuals aged 1 month to 17 years were analyzed. Serum IgG, IgA, and IgM concentrations were measured by rate immunoturbidimetry standardized against international reference materials and evaluated using percentile-based analyses and quantile regression modeling across age-stratified cohorts.

**Results:**

Immunoglobulin concentrations varied substantially across age groups. Median IgG levels increased from 3.65 g/L (2.5th percentile: 2.25 g/L) at 1–3 months to 11.66 g/L (2.5th percentile: 7.69 g/L) at 13–17 years, with parallel age-related increases observed for IgA and IgM. The most pronounced changes occurred during infancy. Although early-life immunoglobulin dynamics did not entirely align with traditional trajectories, they suggested population-specific variations in humoral immune maturation.

**Conclusions:**

This study establishes the first comprehensive, population-specific reference intervals for serum immunoglobulins in Brazilian youth from early infancy through adolescence. By documenting unexpected deviations from traditional early-life trajectories, our study provides a critical benchmark for Brazilian pediatric care and a model framework for Latin American populations.

## 1 Introduction

The human immune system develops during fetal life and undergoes progressive maturation throughout childhood. During the first months after birth, maternally transferred IgG declines while endogenous immunoglobulin production gradually increases, with IgM appearing first, followed by IgG and IgA (1, 2). These age-dependent changes reflect the development of humoral immunity and have direct implications for the interpretation of immunoglobulin measurements in pediatric clinical practice. Accurate age-specific reference intervals are therefore essential to distinguish normal immune maturation from primary and secondary immunodeficiencies (1, 2).

In this context, the interpretation of immunological laboratory parameters, particularly serum immunoglobulin concentrations, must always take into account the patient’s age and stage of immune maturation. Laboratory findings interpreted without consideration of the physiological trajectory of immune development may lead to inaccurate conclusions and potentially inappropriate clinical decisions (1).

Because immunoglobulin concentrations change markedly throughout childhood, age-specific reference intervals (RIs) are essential for accurate pediatric immunological assessment. Percentile-based reference curves capture the dynamic maturation of humoral immunity and provide a more appropriate framework for interpreting laboratory results than fixed reference ranges (3).

Establishing reliable pediatric RIs remains challenging because of the difficulty of recruiting large numbers of healthy children across different age groups. To address these challenges, the Clinical and Laboratory Standards Institute (CLSI) and the International Federation of Clinical Chemistry and Laboratory Medicine (IFCC) recommend rigorous population selection and robust statistical approaches for RI derivation and validation (4).

Despite their clinical importance, population-specific immunoglobulin RIs remain scarce in low- and middle-income countries. Genetic, environmental, nutritional, infectious, and socioeconomic factors may influence immunological parameters, limiting the applicability of reference values derived from European and North American populations. This issue is particularly relevant in Brazil, where laboratory interpretation often relies on external reference standards despite the country’s substantial genetic diversity and population admixture (3).

According to estimates from the Brazilian Institute of Geography and Statistics (IBGE), Brazil currently has approximately 203 million inhabitants, including nearly 45 million residents in the State of São Paulo, one of the most populous and demographically diverse regions in the country (5). These characteristics provide a strong basis for population-based studies conducted within this setting.

Establishing accurate age-specific reference intervals for serum IgG, IgA, and IgM concentrations therefore addresses an important unmet clinical need by providing clinicians with a reliable tool to distinguish normal developmental variation from clinically significant immunological abnormalities. Accordingly, the present study aimed to establish age-specific reference intervals for serum IgG, IgA, and IgM concentrations in healthy children from the State of São Paulo, thereby contributing to improved diagnostic accuracy in the assessment of humoral immunity and supporting evidence-based clinical decision-making in Brazil.

## 2 Materials and methods

### 2.1 Ethical considerations

The study was approved by the Research Ethics Committees of the Federal University of São Paulo (UNIFESP) under approval numbers 3.340.392 and 3.499.511, in accordance with the principles of the Declaration of Helsinki and the regulations of the Brazilian Ministry of Health. Written informed consent was obtained from all legal guardians prior to participant enrollment and blood sample collection.

### 2.2 Study population and recruitment

A total of 2,911 clinically healthy infants, children, and adolescents aged 1 month to 17 years were included. Participants were recruited from well-child outpatient clinics at UNIFESP and the Campinas Municipal Pediatric Outpatient Clinic. Additionally, children undergoing routine preoperative laboratory evaluation before minor elective surgical procedures at these institutions were enrolled.

Recruitment strategies included active engagement of families and educational initiatives designed to improve understanding of the study objectives and the clinical importance of establishing pediatric immunoglobulin reference intervals. These measures facilitated participation and sample collection across all age groups, including young infants.

### 2.3 Inclusion criteria

Eligible participants comprised individuals aged 1 month to 17 years who were clinically healthy at the time of enrollment. Candidates were excluded if they presented with any known medical conditions capable of altering serum immunoglobulin concentrations.

### 2.4 Exclusion criteria

Candidates were excluded from the study if they were older than 17 years or presented with acute or chronic conditions known to influence immunoglobulin levels, including inborn errors of immunity, hematologic disorders, diabetes mellitus, renal or gastrointestinal diseases, malnutrition, or acute inflammatory conditions. Additional exclusion criteria comprised the receipt of blood products within the preceding six months, the regular use of medications (excluding vitamin supplementation), and a nutritional status outside the normal range for age according to the World Health Organization growth standards, encompassing underweight, overweight, and obesity. Finally, individuals were excluded in the absence of written informed consent from a legal guardian.

### 2.5 Sample collection and processing

A single 5-mL peripheral venous blood sample was collected from each participant exclusively for study-related analyses. Specifically, we recruited participants and collected samples between January 2021 and January 2026. Immunoglobulin measurements were performed at the Central Laboratory of Hospital São Paulo, a reference laboratory accredited under ABNT NBR ISO 15189 and the PALC (Laboratory Accreditation Program of the Brazilian Society of Clinical Pathology/Laboratory Medicine). These accreditations ensure adherence to rigorous analytical quality standards, traceability, and reliability of laboratory results (6, 7).

### 2.6 Laboratory analysis

Serum concentrations of IgG, IgA, and IgM were determined using immunoturbidimetry, a widely validated and routinely employed method for immunoglobulin quantification. In this assay, serum samples are mixed with specific antisera, resulting in the formation of insoluble immune complexes. The resulting turbidity, generated by light scattering from these complexes, is measured spectrophotometrically and converted into immunoglobulin concentrations through calibration against standardized reference materials.

All measurements were performed using Roche COBAS 6000 analyzers (Roche Diagnostics International Ltd., Rotkreuz, Switzerland). Raw immunoturbidimetric output obtained in mg/dL was converted to g/L using a standard conversion factor for subsequent statistical analyses.

### 2.7 Statistical analysis

#### 2.7.1 Reference interval determination and partitioning

Demographic characteristics were summarized using absolute and relative frequencies for categorical variables. Sample size was determined by feasibility and aligned with Clinical and Laboratory Standards Institute (CLSI) EP28-A3c guidelines for reference interval studies. Reference intervals were established and verified following these guidelines, including healthy reference population selection, age-specific partitioning, and percentile-based reference limit estimation. Age partitions were defined based on immune system development principles, visual inspection of age-related percentile distributions, and biological plausibility, with particular emphasis on the first year of life due to rapid physiological changes. To assess partitioning appropriateness, immunoglobulin concentrations were compared between adjacent age groups using Welch’s t-test to account for unequal variances. This strategy is conceptually analogous to the Harris–Boyd approach recommended in CLSI guideline EP28-A3c for evaluating the need for separate reference intervals (8).

#### 2.7.2 Statistical testing and validation

As a secondary validation for early infancy, differences between the 1–3-month and 4–5-month age strata were evaluated using the Mann–Whitney U test. Predefined age partitions demonstrating statistically significant differences (p < 0.05) were retained as independent strata to preserve the temporal resolution required to capture rapid developmental changes.

Reference intervals were estimated using descriptive percentile analysis within predefined age categories and quantile regression to generate continuous, smoothed percentile curves across childhood and adolescence. For both methods, the 2.5th, 25th, 50th (median), 75th, and 97.5th percentiles, along with their respective 95% confidence intervals (CIs), were calculated for each immunoglobulin isotype. Quantile regression was selected because it does not assume data normality and is robust for asymmetric biological distributions, such as pediatric serum immunoglobulin concentrations.

#### 2.7.3 Error control and software

Multiple comparisons between adjacent age groups (k = 14 pairwise comparisons) and among self-reported ethnic groups within each age stratum (k = 6 pairwise comparisons) were adjusted using the Bonferroni correction, yielding adjusted significance thresholds of α = 0.0036 and α = 0.0083, respectively. All reported p-values reflect this correction unless otherwise specified. For unadjusted single comparisons, a two-sided p-value < 0.05 was considered statistically significant. Statistical analyses were performed using IBM SPSS Statistics version 20.0 (IBM Corp., Armonk, NY, USA) and Stata version 18 (StataCorp LLC, College Station, TX, USA).

## 3 Results

### 3.1 Study population and demographic characteristics

The study evaluated 2,911 healthy infants, children, and adolescents, consisting of 1,624 (55.8%) males and 1,287 (42.2%) females, aged 1 month to 17 years, with a median age of 4.0 years (interquartile range [IQR], 1.0–9.0 years). Regarding self-reported ethnicity, the majority of participants were White (n = 2,345), followed by mixed-race (n = 511), Black (n = 46), and Asian (n = 9) individuals.

No statistically significant differences in immunoglobulin concentrations were identified among ethnic groups. However, the capacity to detect potential variations within the Black (n = 46) and Asian (n = 9) subgroups was limited by the relatively small sample sizes, which reduced the overall statistical power for these specific comparisons.

### 3.2 Age-dependent immunoglobulin dynamics and partitioning

**Figures 1**–**3** depict the age-related distributions of serum IgG, IgA, and IgM concentrations derived from quantile regression analysis. These smoothed percentile curves illustrate the 2.5th, 25th, 50th, 75th, and 97.5th percentiles plotted alongside individual measurements from the healthy participant cohort. **Tables 1**–**3** present the 2.5th, 25th, 50th (median), 75th, and 97.5th percentiles, including their respective 95% confidence intervals (CIs), for serum IgG, IgA, and IgM concentrations stratified by age group.

**Figure 1:**
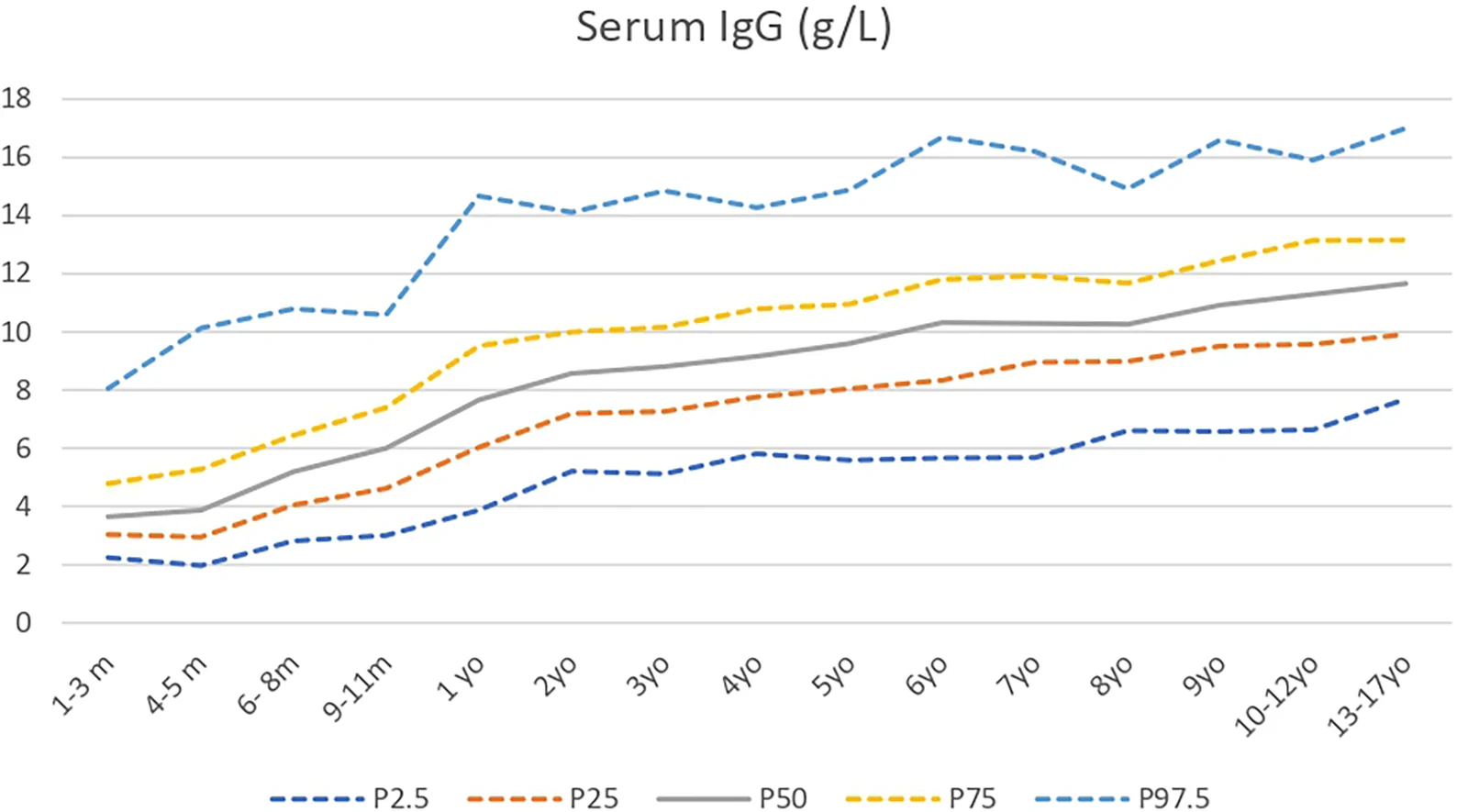
Age-specific percentile curves for serum IgG concentrations (g/L) estimated by quantile regression. Smoothed curves representing the 2.5th, 25th, 50th, 75th, and 97.5th percentiles are shown. Each dot represents an individual measurement obtained from a healthy participant.

**Figure 2:**
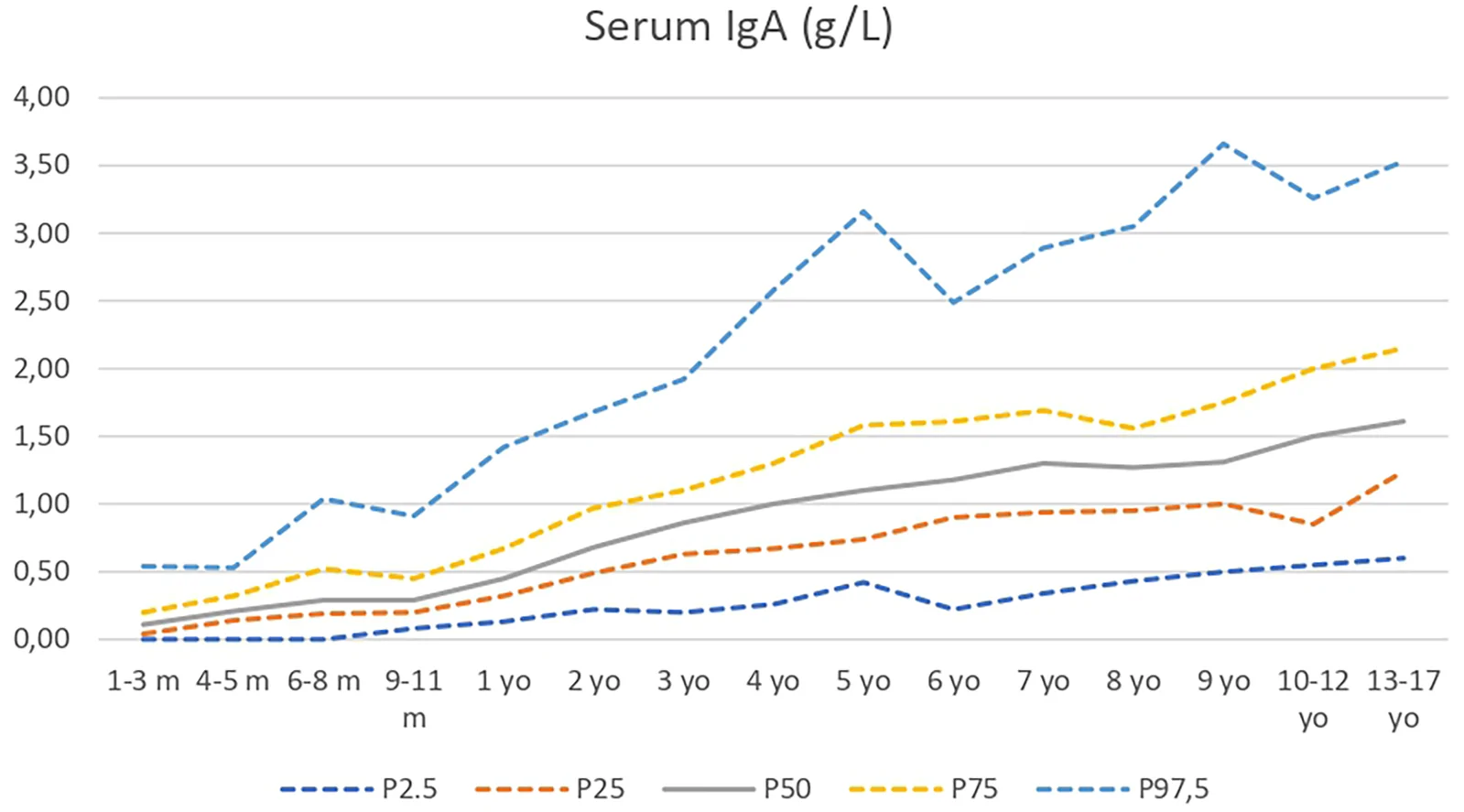
Age-specific percentile curves for serum IgA concentrations (g/L) estimated by quantile regression. Smoothed curves representing the 2.5th, 25th, 50th, 75th, and 97.5th percentiles are shown. Each dot represents an individual measurement obtained from a healthy participant.

**Figure 3:**
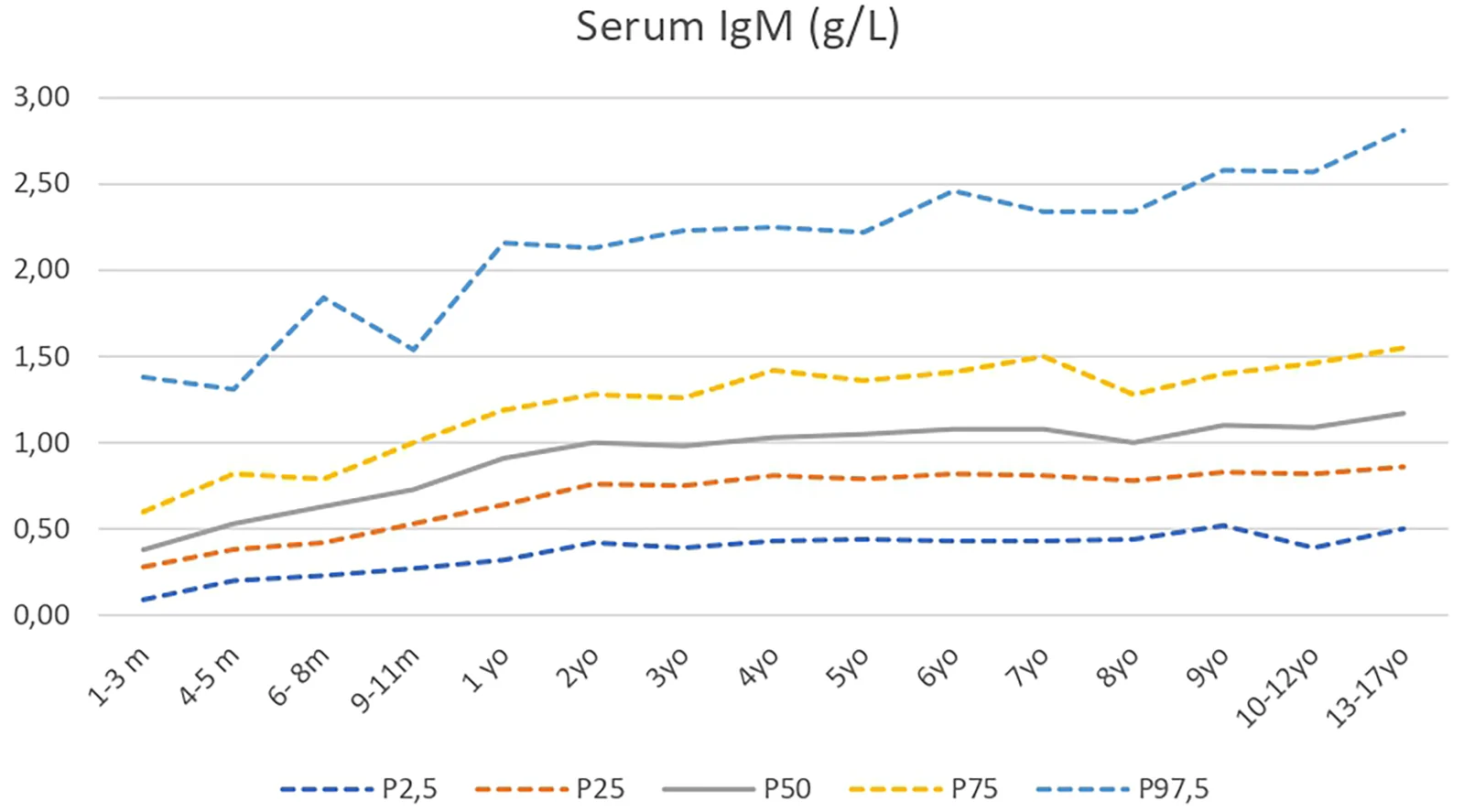
Age-specific percentile curves for serum IgM concentrations (g/L) estimated by quantile regression. Smoothed curves representing the 2.5th, 25th, 50th, 75th, and 97.5th percentiles are shown. Each dot represents an individual measurement obtained from a healthy participant.

**Table 1:** Percentiles of serum IgG (g/L) concentrations according to age group.

Age group (mos/yrs)	P2.5		P25		P50		P75		P97.5		N
	Estimate	95% CI	Estimate	95% CI	Estimate	95% CI	Estimate	95% CI	Estimate	95% CI	
1–3 mos	2.250	2.101–2.399	3.030	2.661–3.399	3.650	3.430–3.870	4.790	4.043–5.537	8.060	7.102–9.018	79
4–5 mos	1.970	1.836–2.104	2.950	2.647–3.253	3.880	3.631–4.129	5.290	4.340–6.240	10.130	8.578–11.682	107
6–11 mos	3.000	2.629–3.371	4.310	4.046–4.574	5.620	5.346–5.894	7.170	6.686–7.654	10.590	10.041–11.139	229
12–23 mos	3.880	3.399–4.361	6.040	5.775–6.305	7.670	7.370–7.970	9.510	9.094–9.926	14.670	13.570–15.770	404
2–7 yrs	5.450	4.949–5.951	7.750	7.604–7.896	9.340	9.191–9.489	10.870	10.655–11.085	15.000	13.186–16.814	1,220
8–12 yrs	6.620	5.819–7.421	9.410	9.181–9.639	10.920	10.574–11.266	12.530	12.126–12.934	15.810	14.983–16.637	526
13–17 yrs	7.690	5.068–10.312	9.920	9.588–10.252	11.660	11.280–12.040	13.160	12.772–13.548	17.000	15.185–18.815	306

Percentiles were estimated using quantile regression analysis. Median values (P_50_) are highlighted in bold. CI, confidence interval; g/L, grams per liter; mos, months; yrs, years.

**Table 2:** Percentiles of serum IgA (g/L) concentrations according to age group.

Age group(mos/yrs)	P2.5		P25		P50		P75		P97.5		N
	Estimate	95% CI	Estimate	95% CI	Estimate	95% CI	Estimate	95% CI	Estimate	95% CI	
1–11 mos	0.000	-0.002–0.002	0.140	0.123–0.157	0.230	0.205–0.255	0.370	0.327–0.413	0.910	0.605–1.215	410
1–6 yrs	0.170	0.137–0.203	0.490	0.469–0.511	0.760	0.730–0.790	1.140	1.093–1.187	2.310	1.359–3.261	1,443
7–8 yrs	0.400	0.252–0.548	0.950	0.884–1.016	1.280	1.209–1.351	1.640	1.509–1.771	2.890	2.532–3.248	254
9–17 yrs	0.550	0.428–0.673	1.140	1.084–1.196	1.500	1.445–1.555	2.000	1.940–2.060	3.420	2.129–4.711	718

Percentiles were estimated using quantile regression analysis. Median values (P_50_) are highlighted in bold. CI, confidence interval; g/L, grams per liter; mos, months; yrs, years.

**Table 3:** Percentiles of serum IgM (g/L) concentrations according to age group.

Age group (mos/yrs)	P2.5		P25		P50		P75		P97.5		N
1–3	0.070	0.020–0.120	0.280	0.232–0.328	0.380	0.302–0.458	0.600	0.463–0.737	1.700	1.410–1.990	74
4 mos–1y	0.270	0.210–0.330	0.530	0.504–0.556	0.750	0.720–0.780	1.040	0.988–1.092	1.950	1.729–2.171	699
2–12 years	0.430	0.372–0.488	0.780	0.759–0.801	1.010	0.985–1.035	1.400	1.366–1.434	2.350	1.100–3.600	1,651
13–17 years	0.500	0.414–0.586	0.860	0.791–0.929	1.170	1.099–1.241	1.550	1.434–1.666	2.810	0.436–5.184	289

Percentiles were estimated using quantile regression analysis. Median values (P_50_) are highlighted in bold. CI, confidence interval; g/L, grams per liter; mos, months; yrs, years.

Collectively, these findings characterize the age-dependent evolution of serum immunoglobulin concentrations in the Brazilian pediatric population. Notably, median IgG, IgA, and IgM concentrations differed significantly between the 1–3-month and 4–5-month age groups (Welch’s t-test, Bonferroni-adjusted p < 0.05 for all comparisons). This statistical divergence justifies and supports the retention of separate, independent age partitions during early infancy to capture rapid physiological changes.

## 4 Discussion

### 4.1 Summary of key findings and novelty

The present study establishes robust reference intervals (RIs) for serum IgG, IgA, and IgM levels in a large cohort of 2,911 healthy children and adolescents from the state of São Paulo, Brazil. To our knowledge, this represents one of the largest pediatric cohorts evaluated for this purpose in Brazil, offering a key reference framework for Latin American populations. The methodological strength of this study derives from the rigorous selection of healthy individuals, detailed age stratification—particularly during the first year of life—and the use of standardized laboratory methodologies.

A major novelty of our findings lies in the observed early-life immunoglobulin dynamics. While our findings align with established principles of humoral system maturation, they challenge the universality of the classic biphasic IgG curve in early life (9–13). Most notably, our cohort demonstrated that the establishment of endogenous synthesis was reflected by a steady, age-related increase in IgG concentrations, rather than the classic biphasic pattern (early decline followed by recovery) traditionally reported in early life (9–13). Recognizing this continuous physiological trajectory is essential to distinguish normal developmental variation from pathological hypogammaglobulinemia during infancy.

### 4.2 Integration into the current understanding of humoral maturation

Our findings regarding IgM and IgA align with the established biological understanding of humoral maturation. Classical studies demonstrated detectable IgM in umbilical cord blood, followed by progressive increases during the first weeks and months of life (11, 13, 14). In parallel, somatic hypermutation has been identified as early as 10 days to 2 months of age, increasing thereafter, while IgM+IgD+CD27+ B cells undergo diversification even in the absence of classical antigen-dependent responses (15). Furthermore, the timing of endogenous IgG production may be influenced by the quantity of maternally transferred immunoglobulin received by the infant (1).

The decline of IgG concentrations during early infancy, largely mediated by the neonatal Fc receptor (FcRn), has been documented as previously described (14). Subsequent recovery reflects the progressive establishment of immunoglobulin synthesis, a key milestone in immune development (11–14).

In our cohort, the establishment of endogenous immunoglobulin synthesis was reflected by a steady age-related increase in IgG concentrations, rather than by the biphasic pattern of early decline followed by recovery traditionally reported in the literature. The steady rise of IgG without a pronounced biphasic nadir. These findings could be explained by a potential artifact of age-binning, sampling resolution, or quantile regression smoothing, rather than suggesting that infants completely bypass maternal antibody decay. While the decline of maternal IgG mediated by the neonatal Fc receptor (FcRn) is a universal physiological event (16), the immediate and efficient onset of endogenous production in this cohort smoothed out the expected traditional decline.

Recognition of these physiological trajectories is essential for distinguishing normal developmental variation from pathological hypogammaglobulinemia during childhood. IgM concentrations increased early in life and subsequently remained relatively stable. Similar patterns have been reported by Lau et al. (17) in Chinese children and by Blanco et al. (18), who related these changes to the developmental distribution of B-cell subsets and plasma cells.

The observation of comparable age-related trends in a large cohort of healthy children, free from disease-related bias, further supports the biological plausibility and external validity of these findings (13). The use of quantile regression for percentile estimation constituted a particularly relevant methodological choice. Unlike models that assume a specific parametric distribution, this approach allows a more accurate representation of the actual distribution of serum immunoglobulin levels across different age groups, thereby enhancing the clinical applicability of the proposed reference intervals (19).

### 4.3 Global and national contextualization

Establishing population-specific RIs addresses a critical, long-recognized gap in Brazilian healthcare, which traditionally relies on European or North American reference values. Data from the CALIPER program confirmed that immunoglobulins are among the pediatric analytes showing statistically significant differences across ethnic groups, making the indiscriminate application of foreign RIs a risk for diagnostic misinterpretation (20).

When compared to international datasets, our age-related trends were largely similar to those reported in Danish (21), Chinese (17), and Iranian (22) cohorts, yet notable differences in absolute values were observed. These variations underscore the impact of genetic and environmental diversity. The extensive background of the state of São Paulo—characterized by centuries of admixture among Indigenous, European, African, and Asian populations—ensures that our sample captures a highly representative proxy of the broader Brazilian pediatric population, distinguishing it from more homogeneous studies (23, 24). Studies such as those by Fuentes-Arderiu et al. (25), conducted in predominantly European Spanish centers, and Kardar et al. (22), based on an Iranian laboratory database, provide important comparative parameters; however, their findings cannot be directly extrapolated to the Brazilian context without prior validation. When compared with data from Løk et al. (21), who evaluated 2,171 Danish children aged 6–18 years, age-related trends were largely similar, although differences in absolute values were observed. Such differences are expected and, as demonstrated by the CALIPER program, reflect ethnic variations relevant to immunoglobulin levels (20). Partially divergent results were also reported by Kanariou et al. (23) in Greek children and by Lau et al. (17) in Chinese children, reinforcing the concept that population-specific reference curves constitute essential diagnostic tools.

Within the Brazilian context, Assis et al. (3) pioneered the application of CLSI methodology in children aged 1–10 years from Cuiabá. However, their study excluded infants under one year of life and was restricted to a single geographic region. Our study builds on this legacy by including infants from the first month of life onward and using quantile regression modeling. Unlike conventional parametric methods, quantile regression allowed an accurate, continuous representation of asymmetric biological distributions without assuming data normality, maximizing clinical applicability (19).

### 4.4 Study limitations and shortcomings

Several limitations should be considered when interpreting these findings. First, the cross-sectional design did not allow longitudinal assessment of individual immune maturation trajectories. Second, although the 1–3-month and 4–5-month age strata included fewer participants (n = 79 and n = 107, respectively) than the minimum of 120 individuals recommended by CLSI EP28-A3c for robust nonparametric percentile estimation, their separation was supported both statistically, by Welch’s t-test (p < 0.05), and biologically, as this period encompasses the steepest decline in maternally derived IgG and the onset of endogenous immunoglobulin production. The corresponding 95% confidence intervals account for the increased uncertainty associated with these sample sizes; therefore, the extreme percentiles (P2.5 and P97.5) in these age groups should be interpreted with caution.

In addition, the limited representation of Asian and Black participants reduced the statistical power of ethnicity-stratified analyses. A methodological artifact was also observed for IgA in the 1–11-month age group, where the lower bound of the 95% confidence interval for the 2.5th percentile was estimated as −0.002 g/L. This negative value resulted from a boundary effect inherent to linear quantile regression when modeling concentrations near zero and does not represent a biologically plausible immunoglobulin level. Accordingly, the interval should be interpreted as effectively bounded at zero, while the corresponding percentile estimate (0.000 g/L) remained unaffected.

### 4.5 Clinical practice and future directions

The inclusion of infants beginning at 1 month of age represents the most distinctive and clinically relevant contribution of the present study, addressing an important gap in the literature regarding reference parameters for humoral immunity during the first year of life. This period is characterized by the rapid decline of maternally derived IgG, the onset of endogenous IgM and IgG production, and persistently low IgA levels. Investigators from the CALIPER initiative (20) and related studies have emphasized that obtaining blood samples from healthy infants represents a major methodological challenge, contributing to the scarcity of normative data in this age group (12, 20–24).

From a clinical perspective, the absence of reference curves for infants directly limits diagnostic precision in the investigation of early-onset primary immunodeficiencies, including X-linked agammaglobulinemia, transient hypogammaglobulinemia of infancy, severe combined immunodeficiencies, and secondary antibody deficiencies. In these scenarios, distinguishing the physiological IgG nadir, which may fall below 300 mg/dL between the third and sixth months of life in healthy infants, from pathological hypogammaglobulinemia critically depends on the availability of age-appropriate reference curves. The present study provides this tool for the Brazilian population for the first time. The principal strengths of this study include the large sample size of 2,911 children, substantially exceeding that of comparable studies, such as Bayram et al. (24), which included 330 Turkish children, and Grunewald et al. (26), which evaluated a smaller cohort; the broad age range from 1 month to 17 years, eliminating the need for complementary external reference tables; the rigorous inclusion criteria ensuring a genuinely healthy population; the use of a consolidated and widely employed laboratory methodology based on immunoturbidimetry.

Obtaining blood samples from healthy infants represents one of the major challenges in pediatric laboratory research. It involves important ethical considerations, particularly in the absence of direct benefit to the participant, as well as discomfort, increased risk of hemolysis, and the need for highly specialized personnel. These factors contribute to the scarcity of published data in children younger than 1 year (20). The strategy adopted in the present study, based on the inclusion of children already undergoing preoperative laboratory testing for minor surgical procedures and children followed in well-child outpatient clinics, combined with active family engagement and educational activities, enabled successful sample acquisition.

The proposed reference curves constitute a robust, biologically grounded, and clinically applicable standard for interpreting serum IgG, IgA, and IgM levels in Brazilian children, with particularly relevant implications for the early diagnosis of primary immunodeficiencies and for monitoring humoral immunity throughout childhood. External validation in independent pediatric cohorts is necessary to confirm the robustness, reproducibility, and generalizability of the proposed age-specific reference intervals. Despite these limitations, the findings presented herein represent an important methodological and scientific advance for pediatric immunology in Brazil.

## Data Availability

The raw data supporting the conclusions of this article will be made available by the authors, without undue reservation.
